# Differences in T-cell infiltrates and survival between HPV+ and HPV- oropharyngeal squamous cell carcinoma

**DOI:** 10.4155/fso.15.88

**Published:** 2016-01-07

**Authors:** Sanne Evelien Matlung, Pauline Maria Wilhelmina van Kempen, Niels Bovenschen, Debbie van Baarle, Stefan Martin Willems

**Affiliations:** 1 Department of Pathology, University Medical Center Utrecht, Heidelberglaan 100, 3584 CX Utrecht, The Netherlands; 2 Department of Otorhinolaryngology, University Medical Center Utrecht, Heidelberglaan 100, 3584 CX Utrecht, The Netherlands; 3 Laboratory of Translational Immunology, University Medical Center Utrecht, Heidelberglaan 100, 3584 CX Utrecht, The Netherlands; 4 Center for Infectious Disease Control, National Institute of Public Health and the Environment (RIVM), Antonie van Leeuwenhoeklaan 9, 3721 MA Bilthoven, The Netherlands

**Keywords:** immune cells, immunology, microenvironment, oropharyngeal cancer, prognosis, T cells

## Abstract

Recent studies have suggested that immune cells as part of tumor's microenvironment could partly explain the better outcome in HPV-associated oropharyngeal carcinoma. We performed a systematic review of the literature focused on differences in immune-infiltrate in HPV+ versus HPV- oropharyngeal cancers. This comprehensive search yielded 4308 original papers, of which 20 satisfied our eligibility criteria. Increase in both circulating and tumor infiltrating CD8+ lymphocytes is mainly seen in HPV+ oropharyngeal carcinoma. Interestingly, the survival benefit associated with increase in immune cells is equal both in HPV+ and HPV- oropharyngeal cancer. Based on these results, our review underscores the role of the immune system in the biological and clinical behavior of oropharyngeal squamous cell carcinomas (OPSCC) and might open doors to further investigate immune modulatory treatment options in OPSCC patients.

## Abstract (lay abstract)

**Lay Abstract:** In the last few years it has become clear that oropharyngeal carcinomas can be caused by an HPV infection. Overall, HPV+ carcinomas have a better outcome. Recent studies suggest that could be partly explained by immune cells in the tumor's microenvironment of HPV+ carcinomas. Following a systematic review of the literature focusing on immunological difference between HPV+ and HPV- oropharyngeal cancers, we found a survival benefit associated with increase in immune cells in both HPV+ and HPV-. We found an increase in CD8+ lymphocytes primarily in HPV+ carcinomas. Our review underscores the role of the immune system in the biological and clinical behavior of these cancers.

**Figure 1.**
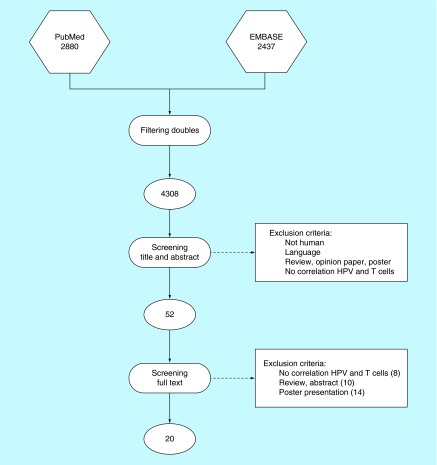
**Flow chart for systematic literature search.**

Head and neck squamous cell carcinoma (HNSCC) is the sixth common cancer worldwide with about 550,000–600,000 new patients per year [1,2]. Despite improvements in diagnostic and therapeutic tools, the 5-year survival rate is still poor, approximately 50% [3]. In addition, all these patients suffer from treatment-induced side effects such as swallowing or speech problems. Therefore, identification of new therapeutic targets based on novel mechanistic studies is urgently needed. Immunotherapy could play a role in the near future, especially in virally induced HNSCC.

HNSCC is a heterogeneous disease originating from multiple different anatomical subsites and depending on the origin varies in biological behavior resulting in varying survival rates and incidences. Traditional risk factors for HNSCC are excessive tobacco and alcohol use [4]. In the last decades, preventive strategies have led to a decrease in HNSCC, mainly of hypopharyngeal and laryngeal SCC. In contrast, there has been a growing proportion of oropharyngeal squamous cell carcinomas (OPSCC) in the USA and Europe in individuals who neither drank nor used tobacco [5]. These were mainly young, white men who are positive for HPV, in particular type 16 [6]. Since the first time that HPV was related to HNSCC (mainly OPSCC) in the early 1980's, there has been an increased interest in the molecular biology of HPV+ OPSCC. HPV infection has been earlier described in the carcinogenesis of the anus, penis, vulva and cervix. Nowadays, 20–80% of OPSCCs are HPV+ depending on geographical location. HPV+ OPSCC presents itself mostly in a higher stage with lymph node metastases compared with its HPV- counterpart [7]. Irrespective of the higher stage at presentation, HPV+ OPSCCs have an improved prognosis [8]. This is thought to be partly related to the expression of wild-type tumor suppressor gene TP53 in HPV+ tumors and absence of field cancerization. Recently, studies showed that the immune system also plays a role in the improved survival of HPV+ OPSCC [9]. This finding has opened the idea that immunotherapy is a possible and less disabling therapeutic option and may be an addition to the current therapy in these patients. To put immunotherapy into practice, better understanding of the exact role of the immune system in HNSCC is needed. Studies have already shown that an increase of immune cells in the tumor and also circulating lymphocytes are associated with a better prognosis and that there is a specific role for T lymphocytes [7]. In this review an overview is given of the differences in T-cell influx between HPV+ and HPV- OPSCC. Unraveling the differences in immune response between HPV+ and HPV- tumors may provide better understanding of the differences in survival between these OPSCCs. Furthermore, it may lead to new treatment modalities such as immunotherapy in, especially, HPV-induced OPSCC. This type of precision therapy hopefully diminishes the unwanted therapy-induced side effects and increases the quality of life of the patients and improves the prognosis of this increasing subtype of HNSCC.

## Materials & methods

### Search strategy

A systematic literature search was conducted in EMBASE and PubMed databases of articles published until September 2015. The research terms were ‘T lymphocytes’ and ‘oropharynx cancer’ with all their synonyms in title and abstracts (Supplementary File 1). After removing duplicates, two authors (PMWK and SEM) independently screened the titles and abstracts of all retrieved records for inclusion, using predefined inclusion and exclusion criteria (see below). Subsequently, full texts of relevant articles were screened for a more detailed selection. A cross-reference search was performed, checking for articles not identified by the original search. Differences in opinion were settled by discussion. Subsequently, the full texts were screened by both authors, which resulted in 20 articles used in this review.

### Study selection

#### Inclusion criteria

The articles were selected based on [1] human studies in English, German or Dutch, [2] the investigated field of HNSCC had to be the oropharynx, [3] containing the correlation between T lymphocytes and HPV status.

#### Exclusion criteria

Studies were excluded when [1] they did not contain original data, [2] it were posters, reviews, opinion papers or abstracts, [3] based on animal studies, [4] the investigated subsite was not the oropharynx, [5] did not describe the relationship between T lymphocytes and HPV status in OPSCC (Figure 1).

#### Data extraction

For the included studies, two authors independently (PMWK and SEM) extracted descriptive data of study population, HPV detection, HPV prevalence and T-lymphocyte infiltration. The following data of included authors were extracted: first author, year of publication, country, tumor location and percentage OPSCC, percentage HPV-positive tumors, HPV detection method, method of T-lymphocyte extraction (peripheral blood or TIL's). To compare the influx or circulation of CD3+, CD4+, CD8+ and FoxP3+ T lymphocytes between patients with HPV-positive OPSCC and HPV-negative OPSCC, p-values were extracted or calculated if sufficient data were provided. Additionally, if reported, the association between T-lymphocyte infiltration or circulation and survival was extracted.

## Results

### Search results

The search retrieved 4308 unique articles (Figure 1). After selection based on title and abstract and subsequent full-text screening, 18 were considered eligible. The main reasons for excluding articles were duplicates, reviews or poster presentations, as well as no correlation described between T cells and HPV-related oropharyngeal carcinomas.

### Study characteristics

The study characteristics of the included studies are summarized in Table 1 [1–20]. In total, the selected 18 studies comprised a total of 1471 patients (range, 17–280 patients per study). Most studies included exclusively OPSCC, but six studies included HNSCC of which 44–92% of the patients had an OPSCC. Fifteen studies were performed in Europe and five in the USA. Although HPV detection methods varied between the included studies, most studies used a combination of p16 immunostaining and PCR. Only Lukeskova *et al*. correlated HPV DNA to HPV mRNA expression. Of the included studies, seven articles investigated the relation between HPV status and circulating T cells, using flow cytometry. Four of these studies also studied the HPV-specific T-cell response and HPV status [1,5,6,11,14,18,19]. The relation between tumor infiltrating lymphocytes (TILs) and HPV status was the subject of research in 12 studies, using immunohistochemistry [2–4,7–10,15–17,20]. In these articles, general T cells were determined by CD3 staining. Furthermore, CD4+, CD8+ and FOXP3+ cells were also stained by their specific antibodies (Supplementary Table 1). Rittà *et al*. also described the association between the viral load and the density of the TILs [2]. Supplementary Table 1 describes the used antibodies for IHC and flow cytometry in the included studies.

#### HPV status & circulating T cells

The extracted outcome data of included six studies are shown in Table 2. Two articles investigated circulating CD3+ T cells in HPV-positive and HPV-negative OPSCC did not show statistically significant differences [14,18]. In addition, one of these articles described a significant decrease in naïve T cells and significant increase of effector T cells and effector memory T cells in HPV+, compared with HPV- OPSCC [18]. Two other articles showed that circulating CD4+ cells were decreased in HPV+ OPSCC, compared with HPV- OPSCC, although this was not significant [5–9,14,18,19]. In contrast, one article showed an increase of CD4+ cells, but again this was not significant [11]. Four of the six articles looked at the circulation of CD8+ cells in OPSCC and HPV status [5,6,11,19]: three articles showed a significantly higher percentage of circulating CD8+ cells in HPV+ OPSCC, taken into account that one article specifically mentions E7_11–20_ epitope as the CD8+ cells target. Also the ratio of CD4/CD8 is significantly lower in HPV+ OPSCC, compared with HPV- OPSCC. Only Heusinkveld *et al*. measured circulating FOXP3 cells and found no statistically significant difference between both groups [1].

#### HPV status & tumor infiltrating lymphocytes

A total of 13 articles investigated the CD3+ tumor infiltrating lymphocytes (Table 2). Two articles showed no differences in overall T-cell infiltrates between HPV+ and HPV- OPSCC [2,20]. However, 5 of the 13 articles showed that CD3+ cells were increased in HPV+ OPSCC, compared with HPV- OPSCC, but in only four articles this effect was significant (p <0.05) [2–4,9,10,15]. Subanalyzing the different T-cell subtypes, eight articles showed an increase in CD4+ TILs in the HPV+ OPSCC, but in only four articles this feature was significant (p <0.05). An increase of CD8+ cells in HPV+ OPSCC was seen in ten articles, but in only six articles the difference was significant [2–4,7–10,13,16,17,20]. In addition, two articles also described the CD4/CD8 ratio as a relevant outcome, concluding that HPV+ OPSCC had a significantly lower CD4/CD8 ratio [2,3]. The FOXP3 T cells were investigated in eight articles, showing a significant increase of FOXP3 in HPV+ OPSCC compared with HPV- OPSCC in only two cases [2–4,7,8,10,13,16].

#### Circulating T cells & survival

Only two of six articles investigating the circulating T cells in OPSCC, described the influence of circulating T cells on the survival [5,19]. The first article showed that elevated Tregs levels were associated with a better overall survival (OS) (p = 0.039). The levels of circulating Tregs were equally distributed in HPV-positive and HPV-negative tumors and therefore not associated with HPV status (p = 0.929). As a whole, patients with elevated Tregs levels and HPV positivity had a significantly better disease specific survival (DSS) and OS. In this same article a significant better DSS was seen in patients with a low CD8+/Tregs ratio (p = 0.012) and this lower feature was independent of HPV status. The second article showed almost the comparable results, looking at CD8+ cells and CD4+ cells. Their results were that a high CD8+ cells and a low CD4/CD8 ratio was associated with a better OS although this was not a significant association [19].

#### Tumor infiltrating lymphocytes & survival

Seventeen studies investigated the relationship between tumor infiltrating lymphocytes and survival (Table 3). Oguejiofor *et al*. concludes that HPV positivity in itself is associated with a significantly better overall survival [10]. Ward *et al*. described in his article that TILs were no prognosticators in HPV- OPSCC, but in HPV+ OPSCC a high number of TILs were related to a significantly better disease-specific survival [7]. In contrast, Kong *et al*. showed a strong HPV+ signal alone that is associated with a significantly better DSS, but that a more intense CD3+ staining has no prognostic value in HPV+ tumors [15]. This same absent prognostic value of CD3+ cells in OPSCC was also concluded by the research groups of Russell *et al*., Badoual *et al*., Oguejiofor *et al*. and Nordfors *et al*. [3,8,10,17]. By not adjusting for HPV status, a high CD3+ staining was associated with a significantly better OS, according to Balermpas and Rajjoub [12,13]. But when relating an intense CD3 staining to the HPV weak/negative group (depending on P16 staining and pyrosequencing signal), 5-year overall survival was significantly better [12,13,15].

Looking at the different T-cell subtypes, CD4+ cells were not significantly related to survival, according to Balermpas, Oguejiofor and Badoual [8,10,13]. In contrast, Nordfors found that high CD4+ is associated with an almost significantly better DFS in HPV- OPSCC [17]. This association was not seen in the HPV+ group, nor seen in the OS in both groups. Looking at high CD8+ cells, Balermpas and Nordfors showed a significantly better overall survival in patients, regardless of HPV status and also Wansom showed this effect according to the DFS [13,17,20]. Jung described that a high CD8α has a better 5-year overall survival and in combination with an HPV+ status they have a better 5-year OS compared with high CD8α+ and HPV- patients [9]. FOXP3 on its own was no significant prognosticator, according to Balermpas and Badoual [8,13]. This was contradicted by Russell and Wansom, who showed that a high number of FOXP3-positive cells were related to a significantly better DFS, regardless of HPV status and OS, adjusted for HPV status [3,20]. In HPV+ and HPV- OPSCC, a higher CD8/FOXP3 ratio is associated with a significantly better cumulative survival [16].

## Discussion & conclusion

In this review, we have examined the differences in T-cell repertoire between HPV+ and HPV- OPSCC. To our knowledge this is the first systematic review investigating the relationship between the prognostic value of immune cells in HPV+ and HPV- OPSCC.

The results of this systematic review should be viewed within the constraints of several limitations. First, studies were performed in various geographical areas with probably variable HPV incidence. Second, the techniques of HPV detection also varied, and due to different sensitivity and specificity, possibly also introduced heterogeneity in HPV status and subsequent correlations. Third, although we included only studies that investigated OPSCC, some also contained tumors from additional subsites. As the effect of immune cells might vary per subsite, it is not certain whether this influenced survival rates. Fourth, antibodies and (both technological and scoring) methods used for detection of immune cells were variable, again introducing (pre)-analytical heterogeneity. Because of this heterogeneity in methods and analyses, statistical pooling was not permitted. Hence, no overall disease-specific survival could be calculated. This warrants the introduction of useful criteria for quality assessment for molecular studies. This is particularly important due to the increase in biomarker studies in the field of personalized cancer care, because these specific molecular biomarkers of tumors including their microenvironment are used for clinical decision-making in the treatment of oncologic patients. We advise caution in interpreting these results because of the small number of candidate genes with overlap and the methodological differences between studies. Comparison and validation of these results was thus impaired. For future research, we recommend adequately designed studies with study populations of explicitly OPSCC, uniform protocols for interpretation of promoter methylation and validation in independent cohorts to evaluate these promising results on a larger scale. Despite this heterogeneity however, most studies point toward an increase in circulating and tumor infiltrating CD8+ lymphocytes in HPV+ OPSCC, but only in half of the cases, this effect is statistically significant. Moreover, this increase in both circulating and tumor infiltrating CD8+ lymphocytes in OPSCC is associated with increased survival. Interestingly this is not only seen in HPV+ OPSCC but also in HPV- OPSCC. The latter might be caused by and increase immunogenicity due to higher mutational load in (alcohol and tobacco induced) HPV- tumors. In general however, this review shows that there is a clear increase in CD8+ circulating and tumor infiltrating T lymphocytes in HPV+ tumors, which is associated with a better survival, independent of HPV status. There is not enough research done in the general increase of circulating lymphocytes (CD3-positive T cells) in OPSCC. This is probably the reason that this effect is not ready statistically significant. In contrast, we do see a significant increase of general tumor infiltrating T cells in HPV+ OPSCC and this has an association with a significantly better survival, although (as in CD8), this effect is independent of the HPV status. This increase in TILs in OPSCC strengthens the idea that circulating lymphocytes are also significantly increased in OPSCC, when the sample size is large enough. The same problem arises in the investigation of circulating CD4- and FOXP3-positive T cells. Although two articles describe a minimal increase of circulating CD4 T cells in HPV- OPSCC compared with HPV+ T cells, this sample size is quite small. Tumor infiltrating T cells have been shown to be increased in HPV-positive OPSCC. In most cases it has no significant role in the survival, regardless of the HPV status. Based on these results, our review underscores the role of the immune system in the biological and clinical behavior of OPSCC and might open doors to further investigate immune modulatory treatment options in OPSCC patients.

## Future perspective

Prognosis of head and neck cancer is still poor and targeted treatment options for oropharyngeal cancer are still limited. Recent studies have shown breakthrough results of immune modulatory therapy in many cancers. As the role of immune cells in HPV-positive and -negative oropharyngeal cancer is being elucidated, immune modulatory therapy could play an important role in the treatment of oropharyngeal cancer.

**Table 1.** **Characteristics of included studies.**

**Study (year)**	**Country**	**Sample size**	**Tumor site**	**Sublocation**	**HPV detection**	**Prevalence HPV**	**Parameters**	**Method**	**Tissue**	**Ref.**
Al-Taei (2013)	UK	13	OPSCC	Tonsil, base of tongue	PCR GP5+6, P16	75%^#^	CD3	FC	PB	[14]
Badoual (2013)	France	64	76.6% OPSCC	?	Inno-Lipa	50%^††^	TIL (CD4, CD8, FOXP3)	IF, FC	FF	[8]
Balermpas (2013)	Germany	101	44% OPSCC	?	P16	28%	TIL (CD3, CD4, CD8, FOXP3)	IHC	FFPE	[13]
Heusinkveld (2011)	Netherlands	21	OPSCC	?	Inno-Lipa	58%^†^	FOXP3	FC	PB	[1]
Hoffmann (2006)	Germany	20	OPSCC	?	HPV16 E7-specific T cells + P16	60% (HV16 E7 specific T cells)	CD8	FC	PB	[6]
Jung (2013)	France	17	OPSCC		PCR	59%	TIL (CD3, CD4, CD8)		FFPE	[9]
Kong (2009)	USA	82	60% OPSCC	?	PCR + P16	44%^‡‡^	TIL (CD3)	IHC	FFPE	[15]
Krupar (2014)	Germany	33	OPSCC	Tonsils (58%), base of tongue	Nested PCR + p16	48%	TIL (CD3, CD4, CD8, FOXP3)	IHC	FFPE	[4]
Lukesova (2014)	Czech Republic	60	92% OPSCC	Tonsils (53%), base of tongue, other	PCR + E6 mRNA expression	42%	CD4, CD8, Tregs	FC	PB	[5]
Näsman (2012)	Sweden	83	OPSCC	Tonsils	PCR + p16	63%	TIL (CD8, FOXP3)	IHC	FFPE	[16]
Nordfors (2013)	Sweden	280	OPSCC	Tonsils (73%) base of tongue	Luminex + p16	79%	TIL (CD4, CD8)	IHC	FFPE	[17]
Oguejiofor (2015)	UK	139	OPSCC	Tonsils (35%), base of tongue	PCR + P16	53%	TIL (CD3, CD4, CD8, FOXP3)	IHC	FFPE	[10]
Partlova (2014)	Czech Republic	45	69% OPSCC	Tonsil (59%), base of tongue	PCR + p16	45%	CD4, CD8	FC	PB	[11]
Rajjoub (2007)	USA	48	OPSCC	Tonsil (69%), base of tongue	CaSki DNA	69%	TIL (CD3)	IHC	FFPE	[12]
Ritta (2013)	Italy	22	OPSCC	?	Nested PCR (FF)	41%	TIL (CD3, CD8, FOXP3)	IHC	FFPE	[2]
Russell (2013)	USA	32	47% OPSCC	?	PCR + P16	26%	TIL (CD3, CD4, CD8, FOXP3)	IHC	FFPE	[3]
Turksma (2013)	The Netherlands	31	OPSCC	?	PCR + P16	36%^¶^	CD3	FC	PB	[18]
Wansom (2010)	USA	47	OPSCC	Tonsils, base of tongue, lateral pharynx	PCR + p16 + mass spectrometry	59%^‡^	CD4, CD8	FC	PB	[19]
Wansom (2012)	USA	50	OPSCC	Tonsils (41%), base of tongue	PCR + mass spectrometry	66%^§^	TIL (CD4, CD8, FOXP3)	IHC	FFPE	[20]
Ward (2014)	UK	274	OPSCC	Tonsils (58%), base of tongue, other	ISH + p16	54%^†^	TIL (CD4, CD8, FOXP3)	IHC	FFPE	[7]

^†^HPV status of two tumors not tested.

^‡^HPV status known in 27 cases.

^§^HPV status known in only 38 cases.

^¶^HPV status known in the oropharyngeal cases with six cases missing.

^#^12 cases DNA for PCR and 13 cases for IHC.

^††^Also HPV18, HPV32, HPV33.

^‡‡^Total group.

FC: Flow cytometry; FF: Fresh frozen; FFPE: Formalin fixed paraffin embedded; IHC: Immunohistochemistry; OPSCC: Oropharyngeal squamous cell carcinomas; PB: Peripheral blood.

**Table 2.** **Results of T-cell influx.**

**Tissue**	**First author**	**CD3**		**CD4**		**CD8**		**FOXP3**		**Ref.**
		**Increased in**	**p-value**	**Increased in**	**p-value**	**Increased in**	**p-value**	**Increased in**	**p-value**	
Peripheral blood	Al-Taei		NS							[14]
	Heusinkveld								NS	[1]
	Hoffmann					HPV+	0.02			[6]
	Lukesova			HPV-	NS	HPV-	NS			[5]
	Partlova			HPV+	NS	HPV+	<0.05			[11]
	Turksma		NS							[18]
	Wansom			HPV-	NS	HPV+	0.04			[20]
Tumor infiltrating lymphocytes	Badoual			HPV+	NS	HPV+	0.01	HPV+	0.059	[8]
	Balermpas		NS		NS		NS		NS	[13]
	Jung	HPV+	<0.001	HPV+	0.032	HPV+	0.006			[9]
	Kong	HPV+	0.03							[15]
	Krupar	HPV+	0.0413	HPV+	NS	HPV+	NS		NS	[4]
	Näsman					HPV+	<0.001	HPV+	<0.001	[16]
	Nordfors			HPV+	0.001	HPV+	<0.001			[17]
	Oguejiofor	HPV+	<0.001	HPV+	<0.001	HPV+	<0.001	HPV+	NS	[10]
	Rajjoub									[12]
	Rittà	HPV+	NS			HPV+	NS	HPV+	NS	[2]
	Russell	HPV+	NS	HPV+	NS	HPV+	NS	HPV+	NS	[3]
	Wansom			HPV+	NS	HPV+	NS	HPV-	NS	[19]
	Ward			HPV+	<0.001	HPV+	<0.001	HPV+	<0.001	[7]

NS: Not significant.

**Table 3.** **Survival.**

**Parameter**	**Study**	**Year**	**Tissue**	**Univariate/HPV status taken into account**	**OS**		**DFS**		**Ref.**
					**HR (p-value)**	**95% CI**	**HR (p-value)**	**95% CI**	
CD3	Balermpas	2013	TIL	HPV-related	(p = 0.007)				[13]
	Balermpas	2013	TIL	Univariate	(p = 0.002)				[13]
	Rajjoub	2007	TIL	Univariate	(p = 0.152)		(p = 0.09)		[12]
CD4	Nordfors	2013	TIL	HPV+related	NS		NS		[17]
	Nordfors	2013	TIL	HPV-related	(p = 0.05)		NS		[17]
	Nordfors	2013	TIL	Univariate	0.990 (p = 0.615)	0.951–1.030	0.991 (p = 0.772)	0.936–1.051	[17]
CD8	Balermpas	2013	TIL	HPV-related	(p = 0.006)				[13]
	Balermpas	2013	TIL	Univariate	(p = 0.002)				[13]
	Lukesova	2014	PB	Univariate	10.023 (p = 0.236)	0.985–1.063	1.027 (p = 1.091)	0.972–1.085	[5]
	Näsman	2012	TIL	HPV+ related			0.27 (p = 0.034)	0.09–0.88	[16]
	Nordfors	2013	TIL	HPV+ related	(p = 0.004)		NS		[17]
	Nordfors	2013	TIL	HPV-related	NS		(p = 0.018)		[17]
	Russell	2013	TIL	Univariate			0.66 (NS)	0.24–1.84	[7]
	Wansom	2012	TIL	Univariate	(p = 0.0137)		(p = 0.0236)		[20]
	Wansom	2014	PB	Univariate	(p = 0.14)				[19]
	Ward	2014	TIL	Univariate	(p = 0.046)				[7]
FOXP 3	Russell	2013	TIL	Univariate			0.44 (NS)	0.15–1.31	[3]
	Wansom	2012	TIL	Univariate	(p = 0.029)		(p = 0.004)		[20]

HR: Hazard ratio; NS: Not significant; PB: Peripheral blood; TIL: Tumor infiltrating lymphocytes.

**Executive summary**

**Background**

The therapeutic options of oropharyngeal carcinoma are fairly limited.
Major causes are smoking, alcohol and HPV.
This makes oropharyngeal cancer highly immunogenic tumors.

**Objective**

The difference in prognostic relevance of CD3+, CD4+, CD8+ and FoxP3+ T lymphocytes between patients with HPV-positive OPSCC and HPV-negative oropharyngeal carcinoma.

**Materials & methods**

Reporting of this review was done systematically.
We conducted a systematic search in the databases of PubMed and EMBASE.
Results were evaluated by two reviewers (SEM and PK) independently.
Review manager 5.3, IBM SPSS 20.0 statistical software was used for the statistical analysis.
Significant heterogeneity was explored through subgroup analysis.

**Prognostic role of immune cells in HPV+ & HPV- oropharyngeal cancer**

Increase in both circulating and tumor infiltrating CD8+ lymphocytes in oropharyngeal cancer is associated with increased survival both in HPV+ OPSCC and also in HPV- OPSCC.
There is a clear increase in CD8+ circulating and tumor infiltrating T lymphocytes in HPV+ tumors, which is associated with a better survival, independent of HPV status.

**Conclusion**

Immune cells play an important role in the biological and clinical behavior of OPSCC.
This opens doors to further investigate immune modulatory treatment in both HPV+ and HPV- oropharyngeal cancer.
