# Supplementary material for: Differences in T-cell infiltrates and survival between HPV+ and HPV- oropharyngeal squamous cell carcinoma
Source: Future Sci OA. 2016 Jan 7;2(1):FSO88. doi: 10.4155/fso.15.88 (PMC5137981; doi:10.4155/fso.15.88)
Supplement: Supplementary file 1 [file fso-02-88-s1.docx]

Supplementary file 1:

Search terms: OPSCC, HNSCC, Head neck squamous cell carcinoma, tumor, tumors, tumour, tumours, carcinoma, carcinomas, cancer, cancers, malignancy, malignancies, neoplasm, neoplasms, SCC, SCCs, oncology, oncological, malignant, malignance, cancerous, base, tongue, otolaryngological, otolaryngologic, otolaryngology, otorhinolaryngology, Glossotonsillar sulcus, Soft palate, oropharynx, oropharyngeal, vallecula, valleculas, tonsil, tonsils, tonsillar, uvula, uvulas, uvulae, palatina arch, base of tongue, Lymphocytes, Lymphocyte, T Lymphocytes, T-Lymphocyte, T-Cells, T Cells, T-Cell, Thymus-Dependent Lymphocytes, Thymus-Dependent Lymphocyte, Thymus-Dependent Lymphocytes, Thymus Dependent Lymphocytes, Thymus-Dependent Lymphocyte, CD4 Positive T Lymphocytes, CD4-Positive T-Lymphocyte, CD4-Positive T-Lymphocyte, CD4-Positive T-Lymphocytes, T4 Cells, T4 Cell, T4 Lymphocytes, T4 Lymphocyte, CD4-Positive Lymphocytes, CD4-Positive Lymphocyte, CD4-Positive Lymphocyte, CD4-Positive Lymphocytes, Helper Inducer T Lymphocytes, Inducer T-Lymphocytes, Inducer T-Lymphocyte, Inducer T-Lymphocytes, Inducer T Lymphocytes, Inducer T-Lymphocyte, Helper-Inducer T-Cells, Helper-Inducer T-Cell, Helper-Inducer T-Cells, Helper Inducer T Cells, Helper-Inducer T-Cel, Helper T-Cells, Helper T Cells, Helper T-Cell, Helper T-Cell, Helper T-Cells, Helper-Inducer T-Lymphocytes, Helper Inducer T Lymphocytes, Helper-Inducer T-Lymphocyte, Helper-Inducer T-Lymphocyte, Inducer Cells, Inducer Cell, Helper T-Lymphocytes, Helper T-Lymphocyte, Helper T-Lymphocytes, Helper T Lymphocytes, Helper T-Lymphocyte, Helper Cells, Helper Cell, Regulatory T Lymphocytes, Regulatory T-Lymphocytes, Regulatory T Lymphocytes, Regulatory T-Lymphocyte, Regulatory T-Lymphocyte, Regulatory T-Cells, Regulatory T Cells, Regulatory T-Cells, Regulatory T Cells, Treg Cells, Treg Cell, Th3 Cells, Th3 Cell, Naturally-Occurring Suppressor T-Cells, Naturally-Occurring Suppressor T-Cell, Tr1 Cells, Tr1 Cell, CD8 Positive T Lymphocytes, CD8 Positive T Lymphocyte, CD8-Positive T-Lymphocyte, CD8-Positive T-Lymphocytes, T8 Lymphocytes, T8 Lymphocyte, CD8-Positive Lymphocytes, CD8 Positive Lymphocytes, CD8-Positive Lymphocyte, T8 Cells, T8 Cell, CD8-Positive Suppressor T-Cells, CD8 Positive Suppressor T Cells, Cytotoxic T-Lymphocyte, Cytotoxic T Lymphocytes, Cytotoxic T-Lymphocytes, Cytotoxic T Lymphocytes, TC2 Cells, TC2 Cell, TC1 Cells, TC1 Cell, Natural Killer T-Cell, Natural Killer T-Cells, NKT Cells, Natural Killer T Cell, NKT Cell, Invariant Natural Killer T-Cells, Invariant Natural Killer T Cells, iNKT Cells, iNKT Cell, Lymphocyte, lymphocytes, Lymphoid Cells, Lymphoid Cell, TcR gamma-delta, TcR gamma delta, gamma-delta T-Cell Receptor, gamma delta T Cell Receptor, gamma-delta T-Cell Receptor, gamma-delta T-Cell Receptors, gamma delta T Cell Receptors, T-Cell Receptor gamma-Chain, T Cell Receptor gamma Chain, T-Cell Receptors delta-Chain, T Cell Receptors delta Chain, T-Cell Receptor delta-Chain, T Cell Receptor delta Chain, T-Cell Receptor delta-Chain.

| Supplementary table 1: **Antibodies and dilutions** | | | | |
| --- | --- | --- | --- | --- |
| **Staining** | **First author** | **Antibody** | **Dilution** | **Cut-off value** |
| CD3 | Al-Taei | eBioscience |  |  |
|  | Balermpas | DAKO, Hamburg, Germany |  |  |
|  | Jung | PC3/188A Santa Cruz, Tebu-bio, Le Perray en Evelines, France | 1:25 | 90% specificity |
|  | Kong | Rabbit monoclonal, Cell Marque, Rocklin, CA |  |  |
|  | Krupar | Neomarkers, Fremont, CA |  |  |
|  | Oguejiofor | Mouse mAb clone F7.2.38 Dako | 1:60 |  |
|  | Rajjoub | Polyclonal rabbit anti-human, Core, Pennsylvania |  | Mean [>1] |
|  | Rittà | Clone PS1, Novocastra, Newcastle Upon Tyne, UK | 1:50 |  |
|  | Russell | Ab5690, Abcam, Cambridge, MA |  |  |
|  | Turksma | Clone SK7, BD Biosciences, Heidelberg, Germany | 1:10 |  |
|  | Ward | Novocastra, Milton Keynes, UK |  |  |
| CD4 | Badoual | Goat anti CD4, R&D | 10μg/ml | Median |
|  | Balermpas | DAKO, Hamburg, Germany |  |  |
|  | Jung | PAB11904, Abnova, Tebu-bio, Le Perray en Evelines, France | 1:25 | 90% specificity |
|  | Krupar | Ventana medical systems, Tucson, AZ |  |  |
|  | Lukesova | Beckmann Coulter, Nyon, Switzerland |  |  |
|  | Nordfors | Clone 1F6, Novocastra Laboratories | 1:40 | Median [13.3] |
|  | Oguejiofor | Clone mAb 4B12 Dako | 1:50 |  |
|  | Partlova | eBioscience |  |  |
|  | Rittà | Clone 1F6, Novocastra, Newcastle Upon Tyne, UK | 1:40 |  |
|  | Wansom 2011 | Ab846, Abcam, Cambridge, MA |  |  |
|  | Wansom | Ab846, Abcam, Cambridge, MA | 1:250 |  |
|  | Ward | Novocastra, Milton Keynes, UK |  |  |
| CD8 | Badoual | Rabbit-anti-CD8 (Abcam) | 1 μg/ml | Median |
|  | Balermpas | DAKO, Hamburg, Germany |  |  |
|  | Hoffmann | BD PharMingen, Heidelberg, Germany |  |  |
|  | Jung | Clone SP16, Abnova, Tebu-bio, Le Perray en Evelines, France | 1:50 | 90% specificity |
|  | Krupar | Ventana medical systems, Tucson, AZ |  |  |
|  | Lukesova | Beckmann Coulter, Nyon, Switzerland |  |  |
|  | Näsman | Clone 4B11, Novocastra Laboratories | 1:40 | Mean |
|  | Nordfors | Clone 4B11, Novocastra Laboratories | 1:40 | Median [36] |
|  | Oguejiofor | Mouse mAb Clone C8/144B Dako | 1:60 |  |
|  | Partlova | Exbio |  |  |
|  | Rittà | Clone C8/144B, DakoCytomation, Denmark | 1:50 |  |
|  | Russell | C8/144B, Dako, Carpinteria, CA |  |  |
|  | Wanson 2011 | VP-C320, Novocastra Laboratories |  |  |
|  | Wansom | VP-C320, Novocastra Laboratories | 1:40 | >24% |
|  | Ward | Novocastra, Milton Keynes, UK |  |  |
| FOXP3 | Badoual | Mouse anti-Foxp3, Abcam, Cambridge, UK | 10μg/ml | Median |
|  | Balermpas | DAKO, Hamburg, Germany |  |  |
|  | Krupar | eBioscience, San Diego, CA |  |  |
|  | Näsman | Clone 236A/E7, eBioscience | 1:100 | Mean |
|  | Oguejiofor | Mouse mAb clone 236A/E7 Abcam, Cambridge UK | 1:40 |  |
|  | Rittà | Clone 236A/E7, eBioscience | 1:100 |  |
|  | Russell | NBPI-43316, Novus, Littleton, CO |  |  |
|  | Wanson | Ab20034, Abcam, Cambridge, MA | 1:200 |  |
|  | Ward | eBioscience, Hatfield, UK |  |  |
